# Comparative perspectives on migration, diversities and the pandemic

**DOI:** 10.1186/s40878-022-00306-z

**Published:** 2022-09-02

**Authors:** Magdalena Arias Cubas, Anju Mary Paul, Jacques Ramírez, Sanam Roohi, Peter Scholten

**Affiliations:** 1 Alfred Deakin Institute for Citizenship and Globalisation, 21 Burwood Hwy, Burwood, VIC 3125 Australia; 2 Sociology and Public Policy, Yale-NUS College Singapore, 16 #01-220, College Ave West, Singapore, 138527 Singapore; 3 Programa de Antropología de Lo Contemporáneo, Facultad de Filosofía, Universidad de Cuenca, Av. 12 de Abril, Cuenca, Ecuador; 4 CeMIS, Georg-August Universtat, Wilhelmsplatz 1, 37073 Göttingen, Germany; 5 Governance of Migration and Diversity, ESSB Faculty, Erasmus University Rotterdam, Burg. Oudlaan 50, 3000 DR Rotterdam, The Netherlands

The global COVID-19 pandemic has challenged social life in profound ways, including migration and migration-related diversities. It has challenged and sometimes deepened existing social structures and inequalities as well as created new ones. However, the precise impact of COVID-19 often remains unclear, as do the broader implications for how we conceptualize and theorize migration and diversities in the field of migration studies. Whereas on the one hand the pandemic showed that global population movements could be constrained at least temporarily, it may also have deepened inequalities as one of the root causes of population movements. And while the pandemic at least temporarily reinforced the national frameworks for inclusion, including but not limited to health, emerging scholarship in the area suggests that it deepened inequalities, exacerbated the access to basic provisions and widened social cleavages within these national frameworks.

The coeval timing of the pandemic and the global BLM movement against racial injustice does not seem to have been just a coincidence. The pandemic brought to the surface and invigorated inequalities and vulnerabilities of specific groups and communities. As amongst others Devakumar et al. (2020) and Wu et al. (2021) have argued, there have been clear racialized consequences of the pandemic. This includes racial minorities’ increased exposure to health risks, reduced access to healthcare and information, their greater involvement in ‘essential’ jobs where home-working was not an option, and their heightened vulnerabilities as a result of the economic consequences of the pandemic.

This special issue seeks to contribute to a better empirical and theoretical understanding of the impact of the pandemic on migration and migration-related diversities. It does so from a purposefully comparative perspective, with studies from across various continents. It is from this comparative perspective that we believe a truly global understanding can be developed of the implications of the pandemic across diverse social, economic and political settings. Also, we believe that from a comparative perspective we can not only enhance a better empirical understanding, but also start to theorize the implications of the pandemic for conceptual and theoretical thinking in migration studies. This will help advance the research field in the context of the emerging risk society in which it is not unimaginable that more similar events will occur in the future.

The special issue (developed from an open and global call to migration researchers) brings 11 original research papers and is structured along four different themes. First, we look at another group that has been affected directly by the pandemic: labour migrants. Secondly, we look at the impact of the pandemic on refugees and asylum seekers, who are amongst the most vulnerable of migrant groups. Thirdly, we look more specifically at migration governance. The pandemic has had a tremendous immediate effect on migration governance, but what more structural challenges and changes can be identified? Finally, we look at inclusion and exclusion. How has the pandemic challenged or added to inequalities in society, and what responses can be identified?

## Research on migration and the pandemic

Although relatively marginal in the field of migration studies, there has been research on the relation between pandemics and migration before COVID-19. Migration scholars have studied the role that international migration played in World War I in the spread of the influenza pandemic of 1918 (Tuckel et al., 2006), and the racialized implications of the AIDS pandemic and the impact of AIDS on mobilities (Marshall, 2005). In general, there has been growing attention in the field to the key topic of health-related migration, but also the health situation of migrants and their access to healthcare.

Since 2020, there have been numerous publications on COVID-19 and migration. Some focus on how the pandemic is challenging and reshaping migration patterns. For instance, Papademetriou and Hooper (2020) show how the pandemic has revealed dilemmas of dependency of various countries that increasingly rely on labour migrants for their economic production but are no longer able to recruit and receive labour migrants during the pandemic. Tagliacozzo et al. (2021) also report that migrant agricultural workers may end up in particularly vulnerable positions during the pandemic. Kluge et al. (2020) observe that refugees also find themselves in particularly vulnerable positions along multiple dimensions, including their access to health care, while being ‘trapped’ in refugee settlements. Bhopal (2020) makes a similar argument regarding undocumented migrants with weak access to health care.

Another important theme in recent studies is how the pandemic will challenge migration governance regimes. Crawley (2021) argues that the pandemic has boosted support for migration restriction and populist resentment against the “other” and thereby also legitimizing a more restrictive approach to the social protection of and service provision to refugees. Newland (2020) also argues that migration governance is likely to change substantially and in more long-lasting ways in response to the pandemic; she predicts that the changes will be even more substantial than in the aftermath of the 9/11 terrorist attacks in the US.

Besides attention to the relation between the pandemic and migration, the impact on migration-related diversities has also been central in various studies. For instance, Clouston et al. (2021) show that there is a clear relation between social inequalities and the spread of COVID-19 in the US; social-economically deprived groups are more susceptible to COVID-19 (in terms of both health and economic impacts), and as a consequence, COVID-19 has deepened pre-existing social inequalities. Crawley (2021) makes a similar argument, and adds that in a post-COVID-19, world refugees are more vulnerable for new forms of migration control and restriction, as well as protection of access to services.

A more specific topic of attention has been discrimination and racism in relation to the pandemic. Various studies, such as Wu et al. (2021), have shown how the pandemic has led to an increase of discrimination against Asian communities. Devakumar et al. (2020) make a similar argument that the COVID-19 pandemic—similar to other pandemics throughout history—contributes to othering and increased fear of marginalized groups. Fear of the increased spread of the virus via international mobility only added further to this othering process. Furthermore, Sabatello et al. (2020) show that the pandemic has had a particular impact on racialized groups, such as black African-Americans.

The contributions from this special issue try to pick up on these important recent contributions in the literature on migration, diversities and the pandemic. In the following, we will briefly discuss the key takeaway points for comparative migration studies from the various articles of this special issue, per topic: refugee migration, labour migration, migration governance and inclusion.

## Labour migration

As employment is one of the key drivers of global international migration, the pandemic has left migrant workers particularly insecure and vulnerable to its vagaries (Cohen, 2020). The interconnections between the labour market, migration governance and healthcare provisions has led to a systemic failure across domains in the COVID-19 aftermath (Tagliacozzo et al., 2021), the ripple effects of which has been felt among migrant workers globally. Even as some crucial forms of migrant labour like seasonal agricultural workers and healthcare workers fall into the ‘essential workers’ category (Fernandes-Reino et al., 2020), their non-integration in host societies is an enduring logic in migration governance (Collins & Bayliss, 2020), prompting a call for a systemic reset (Yeoh, 2020).

In their contribution, Bridget Anderson, Friedrich Poeschel and Martin Ruhs focus on the challenges posed by the pandemic on current systems of labour migration, forcing governments to protect and even expand labour supply to ensure longer term economic resilience in the wake of unpredictable risks and crises. The pandemic has revealed how migrants have transitioned from ‘low skilled workers’ to ‘key workers’ providing various essential services in the host countries. The authors therefore argue for a rethinking of systemic resilience in migrant dependent production and services. Calling for a new agenda for comparative migration research, the authors ask for a thorough assessment of the role transnational labour migration plays across supply chains in building systemic resilience within as well as across systems. Such research can help understand systemic resiliency better and prepare for any future shocks and uncertainties.

In another contribution, Adolfo Sommarribas and Birte Nienaber focus on measures taken by the EU Member States to manage the immigration services in the wake of the pandemic. With COVID-19 turning into an economic crisis, the authors highlight its impact on employment and list out different temporary measures taken by the member states to avoid layoffs and contain rising unemployment including among third country nationals residing in the EU region. One of the early responses by the EU Member States was to close the national borders while intercepting labour supply shortages and preventing third-country nationals from falling into an irregular stay situation. Zooming into the case study of Luxembourg, the authors draw out the multiple responses made by the Grand Duchy to contain the virus spread like barring entry of third country nationals while simultaneously providing relief to temporary workers staying in the country. Despite closing its borders, Luxembourg exempted some migrants including frontier workers, seasonal workers, healthcare professionals and persons involved in the transportation industry (among others) from this ban. These measures were aimed at limiting some of the economic fallouts of the COVID-19 in the country.

Both the articles underline the salience of migrant workers in national and regional economies and while the latter illustrates this point with the empirical examples of temporary changes made to the legal immigration provisions in the EU Member States, the former takes a meso-level approach to argue that the key to systemic resilience oftentimes depend on the supply of migrant labour across the value chain. Given the significance of migrant labour and their continuous supply to the economies of host countries, these papers allude to the challenges COVID-19 posed to the neoliberal non-integrationist logic of migration, suggesting that this logic was temporarily halted or even rebooted during the pandemic.

## Refugee migration

One contribution focuses specifically on how the pandemic has affected refugee migration. In their contribution on refugees agency and coping strategies in refugee camps, Claudia Böhme and Anett Schmitz explore how, forms of agency are being established, made possible or limited within restrictive structures across refugee camps in Germany, Greece and Kenya. Using a combination of on-the-ground and digital ethnography, the authors challenge us to recognise refugees not only as vulnerable to infection, isolation, violence, mistrust and fear, but also as actively engaged individuals with different forms of agency and coping strategies. By highlighting how the specific national contexts, histories and structures of the camps allow or hinder the agency of refugees, Böhme and Schmitz show the agency of refugees in organizing protection themselves as ‘an art of survival’, ranging from resistance and mistrust in Germany, to information, aid and hygiene campaigns in Kenya, to mobilization and protests in Greece.

## Migration governance

On the theme of migration governance at times of the pandemic, Stefan Rother shows that the pandemic has been a challenge to the development of a global migration system, such as the Global Compact of Migration. In response to the pandemic, many countries have chosen their own specifically national and often more restrictive policy paths. However, Rother shows that the pandemic and the ‘zoomification’ of relations in society also provided opportunities in terms of engagement with civil society. This comes both in terms of invited spaces, organized by international institutions, as well as invented spaces where NGOs from all over the world come together to influence global migration governance.

In their analysis of refugee protection during the pandemic, Khangelani Moyo, Kalyango Ronald Sebba and Franzisca Zanker reveal the continued relevance of the national political setting in determining approaches towards refugee protection. They reconstruct how Uganda and South Africa, though facing similar challenges, developed very different policy responses during the first six months of the pandemic. Uganda positioned itself as a refugee protector in order to attract more international humanitarian aid, while South Africa responded instead in a very restrictive way. These authors’ analysis moves beyond a straightforward comparison of refugee policies during a pandemic, to highlight the role played by a given country’s self-image and image management in policy selection. Based on more than 50 in-person interviews and also nine focus groups with refugee and host communities in the two countries in 2020, the authors are able to demonstrate how, even during a pandemic, there is never a political vacuum, which can have serious implications for refugee protection.

Examining migration governance across a broad range of countries (China, Ethiopia, Kyrgyzstan, Moldova, Morocco, Nepal and Thailand), Asel Murzakulova, Mengistu Dessalegn and Neelambari Phalkey argue that the pandemic has created “livelihood insecurities, system insecurities and governance insecurities” that have negatively impacted the position of migrants and their families in the origin country in particular. These insecurities exist around the domains of economics, food, health, and even personal and communal safety. Drawing on mixed methods research in the seven countries listed, the authors review each country’s responses to the pandemic and note the severe strain that various migration systems are now under. At a more mirco-level, the disruptions in migration and remittances have had a profound impact on migrants and migrant-sending households. The uncertainty of migration returning to pre-pandemic levels and the potential of lasting consequences on migrants and migration patterns and pathways, suggests a future of greater risk and exploitation, and a wider gap between formal and informal migration. This paper calls for greater mobility cooperation between countries and suggests strengthening mobility migration frameworks and policies for safer migration and for the rights of migrants.

Overall, all three authors show that government responses to migration during the pandemic tended to be restrictive, and argue that this may continue after the crisis. Investing in national, subnational and regional health security could boost the resilience of migration governance systems for the future.

## Inclusion and exclusion

Finally, this special issue features a number of contributions focusing on the analysis of the inclusion/exclusion. Galstyan and Galstyan focus on the role of social remittances during the pandemic based on their study of Armenian transnational families with migrants in Russia, Belarus and the Czech Republic. They show that "pandemic transnationalism", understood as a form of exchange of information and best practices to cope with the virus, acts in two directions, and is a caring effort between family members who migrated and those who stayed behind. The authors point out that migrants act as intermediaries of ideas and that the information circulating within the family interacts with the pandemic norms stipulated by governments and there is even a greater degree of trust in the information circulating in their own networks.

Anoji Ekanayake and Kopalapillai Amirthalingam show, in their contribution on the economic impact of the COVID-19 pandemic on Sri Lankan migrants in Qatar, that the pandemic has had significant implications for the position of labor migrants, especially wage cuts. Especially low- skilled migrants have faced issues of financial instability and have often been unable to transfer remittances to their home country, nor have they been able to pay their debts or afford food and other basic necessities. This group has had to turn to their relatives in Sri Lanka to send them money, increasing so-called reverse remittances. According to the authors, in terms of migration aspirations, a significant number of migrant workers are considering the possibility of returning permanently to the country of origin.

Asma Khan and Arokkiaraj Heller focus on reverse migration in India, both internal migrants and international migrants who have returned from Gulf countries. In India's pandemic-time economic conditions, significant "involuntary" and "forced" reverse migration has emerged, providing challenges of strategies enabling (or restricting) return and integration. The main problems they face are unemployment, lack of financial support, wage theft and delayed payments, costly repatriation and lack of access to social security. This highlights the insecurities that migrants often face and that surface in the pandemic, as well as the lack of welfare systems to help migrants mitigate these insecurities. This lack of opportunities in their places of origin makes workers think about migrating again once the crisis subsides.

Almina Besic, Andreas Diedrich and Petra Aigner focus on labor market integration support for refugees. They show that in the cases of Austria and Sweden, where a multi-level support structure is in place, the pandemic reinforced already ongoing activities in the field of labour market integration support. The pandemic "further anchored" broader developments, contributed to further mainstreaming and increased work volatility. The authors suggest that developments that were accelerated during the pandemic may over time consolidate as structural approaches towars labour market integration.

Finally, Espinoza et al. argue, in their contribution on social protection systems for migrants and refugees in seven Latin American countries, that the pandemic has triggered specific governance changes. One is that for migrants and refugees, most countries prioritized the provision of basic need and stepped down other measures, which may affect the long-term process of integration. Also, in various cases, pandemic responses came with temporary limitations to exclude migrants based on legal states from social protection, thereby increasing the vulnerability of migrants. The text raises three issues for debate: the so-called "humanitarian crises", "social rights" and "migration governance policies and practices".

## Data Availability

All data and materials used are ours to be used.

## References

[CR1] Bhopal RS (2020). COVID-19: Immense necessity and challenges in meeting the needs of minorities, especially asylum seekers and undocumented migrants. Public Health.

[CR2] Clouston SA, Natale G, Link BG (2021). Socioeconomic inequalities in the spread of coronavirus-19 in the United States: A examination of the emergence of social inequalities. Social Science & Medicine.

[CR3] Cohen JH (2020). Modeling migration, insecurity and COVID-19. Migration Letters.

[CR4] Collins FL, Bayliss T (2020). The good migrant: Everyday nationalism and temporary migration management on New Zealand dairy farms. Political Geography.

[CR5] Crawley H (2021). The politics of refugee protection in a post-covid-19 world. Social Sciences.

[CR6] Devakumar D, Shannon G, Bhopal SS, Abubakar I (2020). Racism and discrimination in COVID-19 responses. The Lancet.

[CR7] Fernández-Reino M, Sumption M, Vargas-Silva C (2020). From low-skilled to key workers: The implications of emergencies for immigration policy. Oxford Review of Economic Policy.

[CR8] Kluge HHP, Jakab Z, Bartovic J, d'Anna V, Severoni S (2020). Refugee and migrant health in the COVID-19 response. The Lancet.

[CR9] Marshall KJ, Urrutia-Rojas X, Soto Mas F, Coggin C (2005). Health status and access to health care of documented and undocumented immigrant latino women. Health Care for Women International.

[CR10] Newland, K. (2020). Will international migration governance survive the COVID-19 pandemic. *MPI Policy Brief, Washington.*

[CR11] Papademetriou DG, Hooper K (2020). Commentary: How is COVID-19 reshaping labour migration?. International Migration.

[CR12] Sabatello M, Burke TB, McDonald KE, Appelbaum PS (2020). Disability, ethics, and health care in the COVID-19 pandemic. American Journal of Public Health.

[CR13] Tagliacozzo S, Pisacane L, Kilkey M (2021). The interplay between structural and systemic vulnerability during the COVID-19 pandemic: Migrant agricultural workers in informal settlements in Southern Italy. Journal of Ethnic and Migration Studies.

[CR14] Tuckel P, Sassler S, Maisel R, Leykam A (2006). The diffusion of the influenza pandemic of 1918 in Hartford, Connecticut. Social Science History.

[CR15] Wu C, Qian Y, Wilkes R (2021). Anti-Asian discrimination and the Asian-white mental health gap during COVID-19. Ethnic and Racial Studies.

[CR16] Yeoh, B. S. (2020). Temporary migration regimes and their sustainability in times of COVID-19. *ILO* (p. 1).

